# Eating Problems in Youths with Type 1 Diabetes During and After Lockdown in Italy: An 8-Month Follow-Up Study

**DOI:** 10.1007/s10880-022-09884-7

**Published:** 2022-05-30

**Authors:** Alda Troncone, Antonietta Chianese, Crescenzo Cascella, Angela Zanfardino, Alessia Piscopo, Serena Rollato, Dario Iafusco

**Affiliations:** 1 Department of Psychology, University of Campania “Luigi Vanvitelli”, Caserta, Italy; 2 Department of the Woman, of the Child and of the General and Specialized Surgery, University of Campania “Luigi Vanvitelli”, Via L. De Crecchio 2, 80138 Naples, Italy

**Keywords:** Type 1 diabetes, Children, Adolescents, Disordered eating behaviors, COVID-19 pandemic

## Abstract

Eighty-five youths with T1D and 176 controls aged 8–19 years were asked to complete online questionnaires (ChEAT and EAT-26) measuring disordered eating behaviors (DEBs) during (baseline) and after (8-month follow-up) the lockdown. DEB symptoms in all participants (especially younger than 13 years), glycemic control, and zBMI were found unchanged from baseline to follow-up (all *p* > .05). After 8 months, the ChEAT/EAT-26 critical score frequency decreased significantly in controls (*p* = .004), as was the score for the ChEAT/EAT-26’s Oral Control subscale in both groups (T1D: *p* = .005; controls: *p* = .01). Participants with T1D, especially those older than 13 years, had higher ChEAT/EAT-26 Dieting scores (*p* = .037) and lower ChEAT/EAT-26 Oral Control scores (*p* = .046) than controls. Unchanged DEB symptoms suggest that the COVID-19 restrictions did not significantly affect participants’ eating behaviors and that a general adaptation to the challenges of lockdown and other pandemic containment measures occurred in both T1D and control participants.

## Introduction

As indicated by evidence from several studies involving young people, living with type 1 diabetes (T1D) has been frequently associated with a higher prevalence of psychopathological symptoms (Buchberger et al., 2016; Delamater et al., 2018; Hagger et al., 2016; Johnson et al., 2013; Reynolds & Helgeson, 2011). The higher vulnerability of individuals with T1D to psychological problems may lead them to experience greater difficulties in managing a stressful condition, such as the COVID-19 pandemic. Some authors have recently stressed how an association between new psychological distress due to COVID-19 and the diabetes-specific psychological problems that are generally experienced could further amplify both psychosocial/interpersonal difficulties and the emotional burden related to diabetes management in individuals with T1D (Mukhtar & Mukhtar, 2020).

Empirical studies have also confirmed that the COVID-19 pandemic exacerbated significant diabetes distress (e.g., coronavirus-specific worries such as greater risk of developing COVID-19, worries about difficulties in glycemic control and diabetes management if infected, and availability of appropriate care) and psychological adaptive difficulties in individuals with T1D (Joensen et al., 2020; Singhai et al., 2020). Evidence collected from adults with T1D during the pandemic has indicated the presence of significant psychological distress, diabetes-related emotional distress, eating disorders, and moderate/severe sleeping disorders (Alessi et al., 2020), as well as significant concern, burden, and mental health disorders in caregivers of youths with diabetes (Alessi et al., 2021).

However, evidence from an Italian study has demonstrated that, despite the stressful psychological conditions of quarantine, children and adolescents with T1D mostly showed high levels of resilience, appropriately complying with the standards of diabetes management, avoiding overeating, regularly engaging in physical activity, and carefully monitoring their glycemic values (Passanisi et al., 2020). Similarly, in a case–control study analyzing the prevalence of eating problems in a sample of Italian youths with T1D during the COVID-19 lockdown (from March 21 to May 3, 2020, a complete lockdown of Italy was ordered by the government as extraordinary measure to combat the spread of the disease), we found that children and adolescents with T1D did not experience more eating problems than a comparison group of age-matched youths without T1D (Troncone, Cascella, et al., 2020; Troncone, Chianese, et al., 2020).

To explore this subject further—especially in light of the significant prevalence of eating problems and their critical impact on diabetes management (Conviser et al., 2018; Goebel-Fabbri et al., 2008; Hanlan et al., 2013; Larrañaga et al., 2011)—we followed the same young patients and re-evaluated them approximately 8 months after the first assessment. Our aim was to explore the prevalence of (symptoms of) disordered eating behaviors (DEBs) across this sample of patients with T1D in comparison to a group of healthy controls during and after the lockdown period in Italy, in order to assess whether DEB symptoms improved, remained stable, or worsened with the easing of the lockdown measures. Metabolic control and zBMI values during the study period were also assessed, and gender and age-related differences were taken into account.

## Method

### Participants and Study Procedure

Participants were patients recruited from the Regional Center of Pediatric Diabetology, University of Campania “Luigi Vanvitelli” Hospital, Naples and healthy controls who were first assessed in April 2020 (Troncone, Cascella, et al., 2020; Troncone, Chianese, et al., 2020). Control participants were recruited from the medical and non-medical friends of the research team. All of the patients with T1D and all of the controls were White and came from the Campania region of Italy. Follow-up data were collected in December 2020 from the same sample. (During the data collection period, Campania was considered a ‘red zone’ for the spread of infection and a high mortality from COVID-19; consequently, containment measures were in place, although they were less stringent than those imposed on the whole country during the spring lockdown—night curfew from 22.00 to 5.00; the closure of gyms, cinema, theaters, swimming pools, coffee places, restaurants, and most shops; movements authorized only for necessity; teleworking strongly encouraged; and home schooling for middle and high schools). In the first evaluation, inclusion criteria were aged 8–19 years; absence of any significant developmental, cognitive, psychological, or medical conditions; T1D diagnosed at least 1 year prior to study enrollment (only for participants with T1D); and use of an intermittently scanned continuous glycemic monitoring (CGM) device for at least 6 months (only for participants with T1D).^1^

The parents of all participants were contacted via phone in order to confirm their continued eligibility and to invite them to participate in the follow-up study. Parents who agreed to participate were sent a link to an online module to complete the consent form and to provide their child’s sociodemographic and clinical data. The link gave their children access to a web-based questionnaire assessing DEB symptoms. The parents of participants with T1D were also asked to provide mean blood glucose values, indicated by the CGM, collected in the 15 and 30 days prior to the study.

Further details about the methodology of the study are described in Troncone, Cascella, et al. (2020), Troncone, Chianese, et al. (2020)).

### Measures

Sociodemographic and clinical data—including age, gender, height, weight, and CGM mean glucose values (only for participants with T1D)—were provided by the parents. In line with evidence that at least 14 days of CGM data is a good estimation of the HbA1c values of individuals with T1D, a current HbA1c values estimate was obtained from the CGM mean glucose values of the previous 4 weeks (a one-month period) (Riddlesworth et al., 2018). Estimated HbA1c values were calculated according to ADAG (A1C-derived average glucose) Study Group data (Klonoff, 2014).

The evaluation of DEBs was carried out by online administration of the following questionnaires: the Children Eating Attitudes Test (ChEAT) (Maloney et al., 1988) for participants aged 8–13 years, and the Eating Attitudes Test-26 (EAT-26) for participants older than 13 years (Garner et al., 1982). ChEAT/EAT-26 are self-report questionnaires designed to evaluate DEB symptoms in the general population. They both consist of 26 items across three subscales: Dieting (13 items), assessing concern with weight and calories, avoidance of fattening foods, and preoccupation with being thin; Bulimia and Food Preoccupation (6 items), measuring thoughts and preoccupations about food and tendencies to binge and purge; and Oral Control (7 items), assessing the degree of self-control over eating and the perceived social pressure to gain weight. Each item is answered on a 6-point Likert scale (from ‘*never*’ to ‘*always*’), with a total score higher than the cutoff of ≥ 20 indicating a presence of symptoms associated with eating problems that require attention and further investigation (Buddeberg-Fischer et al., 1996; Garner et al., 1982). For the present study, the validated Italian versions of both measures were used (ChEAT, Laudadio et al., 2010; EAT-26, Dotti & Lazzari, 1998).

### Statistical Analysis

The statistical analysis was performed with Statistical Package for the Social Sciences (SPSS) version 25.0 for Macintosh. Cronbach’s alpha (α) was computed to assess the homogeneity of the scales. Results were reported in mean ± standard deviation or in absolute and relative frequencies. The relationships among categorical variables were analyzed using chi-square contingency tables. Changes in scores at different time points (baseline to follow-up) of HbA1c, zBMI, and DEB values were conducted separately for patients and controls using repeated measures analyses of variance (ANOVA), with ChEAT/EAT-26 subscale means as a within-subject factor and gender (males vs. females) as a between-subject factor. Comparisons of means between groups (e.g., patients vs. controls, younger vs. older age group) were analyzed with independent samples *t* tests. Age groups were formed following the appropriate age ranges for ChEAT/EAT administration. Results were considered significant at *p* = 0.05 for a two-sided test.

## Results

### Sample Characteristics

The demographic and clinical characteristics of T1D and control participants at both assessments, along with comparisons with non-respondents, are shown in Table 1.

**Table 1:** Clinical sample characteristics at baseline and at follow-up, comparison between participants vs. non-participants with T1D

T1D	Baseline	Follow-up			
	Total sample *N* = 138	Participants *N* = 85	Non-participants *N* = 53	Participants vs. non-participants	
	*M* (*SD*)	*M* (*SD*)	*M* (*SD*)	*test*	*p*
Gender (*n*) (male/female)	65/73	35/50	29/24	χ^2^ = .106,	.745
Age	13.67 (3.21)	13.81 (3.07)	15.44(3.1)	*t*(136) = -4.147	< .0001
Diabetes duration (years)	5.98 (3.22)	6.62 (3.15)	6.4(3.55)	*t*(136) = -.900	.370
HbA1c (%)					
Estimate 15 days	8.45 (1.44)	8.56(1.68)	–	–	–
Estimate 30 days	8.42 (1.33)	8.61 (1.56)	–	–	–
Latest visit	8.24 (1.2)	–	8.35(1.34)	*t*(133) = -.909	.365
zBMI					
Current^a^	.53 (1.01)	.47 (.93)	–	–	–
Latest visit	.89 (1.03)	.77 (.96)	.96(1.01)	*t*(130) = -.523	.602

Data are presented as mean values and standard deviations unless otherwise stated. Dash is inserted when it is not possible to obtain the data. T1D = type 1 diabetes; *N* = number of subjects; zBMI = standardized body mass index

^a^Self-reported values

Of 138 children and adolescents with T1D (aged 8.01–19.11) who were initially assessed, 85 (aged 8.08–19.11; 35 male) were re-enrolled and 53 refused (aged 9.04–20.05; drop-out rate: approx. 38%); of 276 healthy controls who were initially assessed, 176 (73 male) participated in the follow-up and 100 did not (drop-out rate: approx. 36%). For participants with T1D, the main refusal reasons were patients had switched from continuous subcutaneous insulin infusion to multiple daily injection therapy (*n* = 6); were no longer using a CGM device (*n* = 7); could not be reached by phone (*n* = 28); and were no longer interested in the research topic (*n* = 12). For control participants, 30 could not be reached, 24 were unwilling for their children to undergo psychological evaluation again, 32 showed a lack of interest, and 13 were busy with other activities; 1 was excluded after recruitment because he had a genetic illness.

The non-participating patients with T1D (*N* = 53) did not differ from those who participated in terms of distribution of gender (19 m/24 f), duration of illness, HbA1c, or zBMI, but they were older than participants (Table 1).

### DEBs from Baseline to Follow-Up

The mean values of changes in the variables of interest over the 8-month study period are shown in Table 2. The Cronbach’s alpha for ChEAT/EAT-26 total and subscale scores demonstrated adequate internal consistency (Total score α = 0.852; Dieting α = 0.814; Oral control α = 0.661; Bulimia food preoccupation α. = 707).

**Table 2:** Clinical Data, ChEAT/EAT-26 Mean Scores of T1D, and Control Participants at Baseline and Follow-up

	T1D				Control				T1D vs. control			
	Baseline *N* = 85	Follow-up *N* = 85			Baseline *N* = 176	Follow-up *N* = 176			Baseline		Follow-up	
	*M* (*SD*)	*M* (*SD*)	*test*	*p*	*test*	*p*			*test*	*p*	*test*	*p*
ChEAT/EAT-26												
Score ≥ 20% (*n*)	9.4 (8)	12.9(11)	χ.^2^ = 0.5333	.46	10.2(18)	11.4(20)	χ.^2^ = 0.118	.73	χ.^2^ = 0.0425	.837	χ.^2^ = 0.1363	.712
Dieting	6.42 (5.51)	6.78 (6.4)	*F*(1,83) = .301	.584	5.02(5.72)	4.99(5.83)	*F*(1,174) = .020	.889	*t*(259) = 1.875	.062	*t*(259) = 2.241	.026
Oral control	2.53 (2.39)	1.85(2.31)	*F*(1,83) = 6.027	.016	2.57(3.37)	2.36(3.35)	*F*(1,174) = .616	.434	*t*(259) = -.107	.915	*t*(259) = -1.452	*.148*
Bulimia food preocc	1.26 (2.05)	1.47(2.45)	*F*(1,83) = .483	.489	1.20(2.21)	1.28(2.5)	*F*(1,174) = .035	.851	*t*(259) = .210	.834	*t*(259) = .586	.558
Total score	10.21 (7.4)	10.09(8.82)	*F*(1,84) = .021	.884	8.79(8.38)	8.64(9.5)	*F*(1,174) = .021	.884	*t*(259) = 1.333	.184	*t*(259) = 1.184	.237
HbA1c (%)												
Estimate 15 days	8.49 (1.33)	8.56(1.68)	*F*(1,83) = .126	.724	–	–	–	–	–	–	–	–
Estimate 30 days	8.51 (1.31)	8.61 (1.56)	*F*(1,83) = .496	.483	–	–	–	–	–	–	–	–
Current zBMI^a^	.46 (1.02)	.47 (.93)	*F*(1,83) = .045	.832	.45 (.99)	.28(1.06)	*F*(1,174) = 3.128	.079	*t*(259) = .106	.915	*t*(259) = 1.410	.160

Comparisons of Means and Frequencies at Baseline vs. Follow-up

Data are presented as mean values and standard deviations unless otherwise stated. Dash is inserted when it is not possible to obtain the data. T1D = type 1 diabetes; *N* = number of subjects; zBMI = standardized body mass index; Bulimia food preocc. = Bulimia and Food Preoccupation

^a^Self-reported values

#### T1D Sample

At the follow-up evaluation, no significant changes were found in the Hb1Ac levels or in zBMI values in the T1D sample (Table 2), with no gender differences (HbA1c estimate 15 days *F*(1,83) = 0.214, *p* = 0.645; HbA1c estimate 30 days *F*(1,83) = 0.212, *p* = 0.647; zBMI *F*(1,83) = 0.102, *p* = 0.751). During the 8-month study period, all ChEAT/EAT-26 scores remained stable (score ≥ 20 frequency; Dieting; Bulimia, Total score) except the Oral Control subscale, the scores of which were found to be significantly decreased (Table 2). In all comparisons of ChEat/EAT-26 subscale scores except for Oral Control (*F*(1,83) = 0.082, *p* = 0.775), female participants scored significantly higher than male participants (Dieting *F*(1,83) = 7.736, *p* = 0.007; Bulimia *F*(1,83) = 7.711, *p* = 0.007; Total score *F*(1,83) = 8.828, *p* = 0.004).

In terms of age, in participants with T1D who were both younger and older than 13 years, HbA1c, zBMI values, and ChEAT/EAT-26 scores were confirmed as unchanged at the follow-up. However, in participants with T1D who were younger than 13 years, Oral Control scores were found to be lower at follow-up (*n*^2^ = 0.196), although in older ones they were found to be unchanged (Table 3).

**Table 3:** ChEAT/EAT-26 Mean Scores of T1D and Control Participants Grouped by Age at Baseline and Follow-up

	Type 1 diabetes									
	Age ≤ 13 n = 38				Age > 13 n = 47				Age ≤ 13 vs. > 13	
	Baseline	Follow-up			Baseline	Follow-up			Follow-up	
	M (SD)	M (SD)	test	p	M (SD)	M (SD)	test	p	test	p
ChEAT/EAT-26										
Score ≥ 20% (*n*)	5.3(2)	2.6(1)	χ.^2^ = 0.347	.55	12.8(6)	21.3(10)	χ.^2^ = 1.2051	.27	χ.^2^ = 6.483	.001
Dieting	5.50(4.39)	4.48(4.55)	*F*(1,37) = .607	.441	7.17(6.21)	8.34(7.26)	*F*(1,46) = 1.374	.247	*t*(83) = -2.588	.008
Oral control	3.61(2.45)	2.39(2.71)	*F*(1,37) = 9.024	.005	1.66(1.97)	1.4(1.85)	*F*(1,46) = .478	.493	*t*(83) = 1.922	.059
Bulimia food preocc	1.08(1.44)	.95(1.68)	*F*(1,37) = .202	.656	1.40(2.44)	1.89(2.88)	*F*(1,46) = 1.775	.189	*t*(83) = -1.893	.062
Total score	10.18(5.42)	8.18(6.64)	*F*(1,37) = 3.515	.069	10.23(8.74)	11.64(10.06)	*F*(1,46) = .915	.344	*t*(83) = -1.897	.061
HbA1c (%)										
Estimate 15 days	8.68(1.34)	8.89(1.69)	*F*(1,37) = 1.122	.296	8.35(1.33)	8.29(1.64)	*F*(1,46) = .049	.826	*t*(83) = 1.652	.102
Estimate 30 days	8.64(1.26)	8.91(1.62)	*F*(1,37) = 2.195	.147	.84(1.36)	8.37(1.48)	*F*(1,46) = .019	.865	*t*(83) = 1.609	.111
Current zBMI^a^	.26(1.36)	.39(1.21)	*F*(1,37) = .679	.415	.62(.62)	.54(.63)	*F*(1,46) = 1.946	.170	*t*(83) = -.685	.497

	Controls									
	Age ≤ 13 *n* = 74				Age > 13 *n* = 102				Age ≤ 13 vs. > 13	
	Baseline	Follow-up			Baseline	Follow – up			Follow-up	
	*M (SD)*	*M (SD)*	*test*	*p*	*M (SD)*	*M (SD)*	*test*	*p*	*test*	*p*
*ChEAT/EAT-26*										
Score ≥ 20% (*n*)	9.5(7)	5.4(4)	χ.^2^ = 8.115	.004	10.8(11)	15.7(16)	χ.^2^ = 1.252	.263	χ.^2^ = 4.500	.034
Dieting	3.95 (4.08)	3.81(4.69)	*F*(1,73) = .044	.834	5.8(6.58)	5.85(6.42)	*F*(1,101) = .003	.953	*t*(174) = -2.438	.016
Oral control	3.89(4.1)	2.5(3.37)	*F*(1,73) = 7.092	.010	1.61(2.3)	2.26(3.34)	*F*(1,101) = 3.146	.079	*t*(174) = .459	.647
Bulimia food preocc	1.03(1.84)	.72(1.39)	*F*(1,73) = 1.993	.162	1.32(2.45)	1.69(3.01)	*F*(1,101) = 1.018	.315	*t*(174) = -2.868	.005
Total score	8.86(6.35)	7.03(7.12)	*F*(1,73) = 3.525	.064	8.74(9.62)	9.8(10.86)	*F*(1,101) = .656	.420	*t*(174) = -2.046	.042
*HbA1c (%)*										
Estimate 15 days	–	–	–	–	–	–	–	–	–	–
Estimate 30 days	–	–	–	–	–	–	–	–	–	–
Current zBMI^a^	.15(1.07)	.22(1.12)	*F*(1,73) = .229	.633	.66(.88)	.33(1.02)	*F*(1,101) = 10.094	.002	*t*(174) = -.703	.483

	Type 1 diabetes vs. control			
	Age ≤ 13		Age > 13	
	Follow-up		Follow-up	
	*test*	*p*	*test*	*p*
ChEAT/EAT-26				
Score ≥ 20% (*n*)	χ.^2^ = .453	.501	χ.^2^ = .698, .403	.403
Dieting	*t*(110) = 1.112	.268	*t*(147) = 2.109	.037
Oral control	*t*(110) = -.167	.868	*t*(147) = -2.016	.046
Bulimia food preocc	*t*(110) = .776	.439	*t*(147) = .397	.692
Total score	*t*(110) = .833	.407	*t*(147) = .980	.329
HbA1c (%)				
Estimate 15 days	–	–	–	–
Estimate 30 days	–	–	–	–
Current zBMI^a^	*t*(110) = .755	.452	*t*(147) = 1.528	.129

Comparisons of Means and Frequencies at Baseline vs. Follow-up

Data are presented as mean values and standard deviations unless otherwise stated. Dash is inserted when it is not possible to obtain the data. T1D = type 1 diabetes; *N* = number of subjects; zBMI = standardized body mass index; Bulimia food preocc. = Bulimia and Food Preoccupation

^a^Self-reported values

#### Control Group

In control participants, no changes were observed from baseline to follow-up in zBMI values, with no gender differences (*F*(1,174) = 0.001, *p* = 0.970), nor were changes observed in ChEAT/EAT-26 scores (all *p* > 0.05) (Table 2). In all comparisons of ChEAT/EAT-26 subscale scores, female participants scored significantly higher than male participants (Dieting *F*(1,174) = 23.678, *p* < 0.0001; Oral control *F*(1,174) = 7.508, *p* = 0.007; Bulimia *F*(1,174) = 7.506, *p* = 0.007; Total score *F*(1,174) = 25.346, *p* < 0.0001).

Additionally, in terms of age, no changes were observed in control participants younger than 13 years in zBMI values and in most ChEAT/EAT-26 subscale scores (Dieting; Bulimia; Total score). Control participants older than 13 years showed increased zBMI but no changes in ChEAT subscale scores (Dieting; Bulimia; Total score). Only in participants younger than 13 years were the frequency of ChEAT/EAT-26 total score ≥ 20 and the Oral Control score (*n*^2^ = 0.089) found to be decreased at follow-up, while in participants older than 13 years they were found to be unchanged (frequency of ChEAT/EAT-26 total score ≥ 20; Oral Control score) (Table 3).

### DEBs in T1D GROUP Versus Control Group

In line with what was reported in the original sample (Troncone et al., 2020a, Troncone, Chianese, et al., 2020), at the baseline no differences were found between T1D and control participants in terms of zBMI values and ChEAT/EAT-26 scores (Table 2).

On comparing T1D versus control participants after 8 months, no differences were observed in zBMI or most ChEAT/EAT-26 scores (frequency of total score ≥ 20; Oral control; Bulimia; Total score). Only Dieting scores were found to be higher in participants with T1D than in control participants (Table 2). In terms of age group, at follow-up, only participants with T1D who were older than 13 years had Dieting scores that were significantly higher and Oral Control scores that were significantly lower than control participants (Table 3).

### DEBs in Participants Younger Versus Older Than 13 Years

#### T1D Group

On comparing follow-up of participants with T1D who were younger and older than 13 years, those older than 13 years more frequently reported an EAT-26 total score ≥ 20 and scored higher in the Dieting ChEAT/EAT-26 subscale than younger participants (Table 3).

#### Control Group

On comparing follow-up of control participants younger and older than 13 years, those older than 13 years more frequently reported an EAT-26 total score ≥ 20 and scored higher on the ChEAT/EAT-26 subscales (Dieting; Bulimia; Total score) than younger participants (Table 3).

## Discussion

In recent studies examining the psychological impact of the COVID-19 pandemic, there has been limited evaluation of DEB rates in youth with T1D, apart from describing negative effects on eating habits in terms of eating more/less than usual, struggling to maintain a healthy balanced diet (Agarwal et al., 2020; Fisher et al., 2020) and significant impacts on general nutritional behavior (Grabia et al., 2020). Together with the previous investigation conducted with the original sample (Troncone, Cascella, et al., 2020; Troncone, Chianese, et al., 2020), the present study was the first to evaluate eating problems experienced by youths with T1D during and after lockdown in Italy. As such, it contributes to the literature by expanding the existing knowledge about the prevalence of DEBs in youths with T1D, examining potential changes in eating behaviors over an 8-month period in the extraordinarily challenging time of the COVID-19 pandemic.

The present results suggest that after 8 months, despite the enduring demands exacted by COVID-19 restrictions, DEB symptoms in both T1D and control participants were found to be mainly unchanged, especially in participants older than 13 years. In addition, no changes in glycemic control or in zBMI values were observed in T1D participants, while in controls, zBMI was found to have actually decreased. In particular, in terms of HBA1c, even though glycemic control values were found to be higher than the American Diabetes Association’s recommended values (American Diabetes Association [ADA], 2020), they were largely analogous to population data in similar age groups (Clements et al., 2016; National Paediatric Diabetes Audit [NPDA], 2016). In line with data on individuals with and without T1D (Baechle et al., 2014; Colton et al., 2015; Neumark-Sztainer et al., 2011; Wisting et al., 2013), female participants had higher DEB scores than male participants in both the T1D and control samples.

From baseline to follow-up, a significant change was observed only for participants younger than 13 years—with and without T1D—in the degree of self-control around eating, which decreased. In control participants younger than 13 years, the frequency of critical ChEAT/EAT-26 scores were also lower at follow-up. The tendency to perceive self-control around eating was found to be lower in participants with T1D who were older than 13 years than it was in matched controls.

To this end, we assume that the need to control hunger and to avoid food if hungry due to fearing weight gain was probably augmented by the changes in lifestyle related to COVID-19; thus, reduced oral control after the lockdown period leads us to believe that a general adaptation to the pressure and challenges imposed by pandemic containment measures has been occurring in all participants, with and without T1D. The lack of an increase in zBMI values—commonly considered an indicator of nutritional status and overall health (DuBose et al., 2015; Nuttal, 2015)—can be considered a further indication of effective management of COVID-related problems, especially problems that stimulate daily routines modifications that trigger aberrant dietary habits and consequent weight gain. Accordingly, it should be noted that evidence recently collected from Italian individuals with T1D during the COVID-19 pandemic also indicates good diabetes management and improvement in several indicators of glucose control, despite the limitations and difficulties due to the pandemic (Longo et al., 2020).

During the lockdown period (Troncone, Cascella, et al., 2020; Troncone, Chianese, et al., 2020), participants with T1D did not show substantial differences in eating problems compared to control participants. Participants with T1D, especially those older than 13 years, differed from controls in eating behavior only in showing higher dieting attitude and concern with weight and calories. This higher attention to avoiding fattening foods and preoccupation with being thin can possibly be considered as an expected result of the constant attention to diet, weight, food, and the dietary regimen that individuals with T1D typically need to maintain for optimal daily diabetes management. In contrast to what was found at the baseline, after 8 months, participants older than 13 years—with and without T1D—showed higher DEB frequencies and symptoms than younger participants. However, it is worth mentioning that a tendency for EAT scores to increase with age has been previously described (Garfinkel & Newman, 2000) and confirmed by longitudinal studies reporting rising ChEAT scores with age during adolescence, especially in girls (Ferreiro et al., 2021; Wong et al., 2011).

In interpretations of the present findings, it should be also noted that during the data collection period, the schools in Italy were still closed and children and adolescents were staying at home. Given the assumption that adolescence is a time of difficult transition (e.g., transition into secondary school) and the demanding process of maturation toward adulthood, it can be hypothesized that extensive physical distancing, digital school attendance, limitation of sports and outdoor activities, social restrictions and isolation, and overuse of the Internet and social media may have more strongly negatively impacted adolescents’ lives and affected their everyday behaviors.

Several authors (Buzzi et al., 2020; Guessoum et al., 2020; O’Sullivan et al., 2021; Zhang et al., 2020) have recently stressed how the COVID-19 pandemic and quarantine pose a tough challenge to adolescent psychological health, affecting emotions and relationships with peers and parents; it has harmfully impacted mental health and stimulated maladaptive behaviors. Accordingly, adolescents were described as suffering from more anxiety symptoms than children (Duan et al., 2020), and sadness was found significantly more frequently in older/adolescent age groups than in younger ones (Esposito et al., 2021).

Overall, the present results should be viewed in light of the premise that stressful conditions, such as the COVID-19 pandemic, may further exacerbate the psychological conditions of those showing higher vulnerability to psychological problems/stressors, be it chronically ill individuals (Wang et al., 2020) or individuals suffering from mental health problems (Fiorillo & Gorwood, 2020; Yao et al., 2020). From this perspective, individuals with T1D have a higher risk of developing psychological comorbidities and DEBs, given the specific and complex nature of the social and psychological needs of these children (especially during a pandemic) (Conviser et al., 2018; Hagger et al., 2016; Reynolds & Helgeson, 2011; Troncone, Cascella, et al., 2020; Troncone, Chianese, et al., 2020; Young et al., 2013). Therefore, the lack of significant changes in eating problems from lockdown to follow-up may indicate the success of diabetes care and management and suggest that COVID-19 restrictions did not significantly affect the eating behaviors of our clinical sample, despite HbA1c values being slightly higher than target values for this age (ADA, 2020).

In addition, it should be taken into account that during the lockdown, in order to meet patients’ needs, telemedicine care activities were implemented by the center’s staff, and many patients were seen and helped via video-consultation and by the use of communications technologies (e.g., telephone, email, smartphone applications, and computers). In line with evidence demonstrating the application of telemedicine as a useful support in managing diabetes (Farmer et al., 2005; Frielitz et al., 2020), we can speculate that the use of communications technology enabled interactions between patients and their doctors, which positively impacted the day-to-day lives of young patients with T1D and their families and reduced the risk of developing new psychological conditions or worsening pre-existing ones.

The interpretations suggested here will remain speculative, and several limitations in the current study need to be acknowledged. To start, data were collected through online questionnaires, so the answers may have been influenced by the participants’ degree of understanding and cooperation. Such a data collection method may have yielded imprecise anthropometric, clinical, and psychological data (e.g., height, weight, and blood glucose levels as reported by parents). Moreover, no assessment was made of a number of relevant variables, such as SES, educational attainment, and computer/smartphone literacy; this prevented controlling for the variables’ possible confounding effects on the findings (e.g., their possible role in influencing participation in an online study as a result of inequality in Internet access). Additionally, the sample size was relatively small and participants came from the same region and from one diabetes center, limiting the generalization of the results. In addition, recruitment procedures based on voluntary participation may have produced sampling bias. Furthermore, despite the fact that the comparison of the clinical characteristics of participants and non-participants revealed no significant differences, the refusal rate was relatively high. Similarly, a further limitation to the generalizability of the study was the exclusion of youths with T1D with mental health symptoms such as anxiety, depression, or emotion or behavior regulation difficulties—who are likely to also be more vulnerable to DEBs. The exclusion of participants with cognitive, developmental, and psychological problems did not allow an assessment of DEBs among participants with mental health problems, which could have been also useful for reaching a deeper comprehension of the psychological conditions of youths while facing COVID pandemic challenges. Finally, it is also important to recognize that our assessment of DEBs was based on only one measure which was designed for the general population; thus, it may have failed to identify diabetes-specific DEBs (i.e., insulin omission) or may have misidentified healthy self-management behaviors of T1D as disordered habits (e.g., constantly monitoring and selecting food, dietary restraints, etc.).

It would be interesting if a more complete analysis in future studies examined DEBs by using an interview-based assessment that allows for deeper evaluation of eating problems and diabetes-specific unhealthy eating habits in individuals with T1D. Further investigations that more accurately evaluate both psychological adjustments and psychopathological conditions (such as DEBs or co-occurring psychological disorders) are also necessary to fully understand the psychological impact of the COVID-19 pandemic on individuals with T1D. Furthermore, it should be noted that some recent evidence suggests a significant exacerbation of food insecurity (FI) during the pandemic (Parekh et al., 2021). Specifically, according to a recent Italian study, FI risk was found to be particularly high in lower-income households living in Southern Italy (Dondi et al., 2021). Given that FI has been described as associated with eating disorders (Hazzard et al., 2020) as well as with adverse physical outcomes that may further complicate diabetes management (Daly et al., 2021), worries about food access should be also assessed in future studies, in order to more fully understand its possible contribution to eating problems and DEBs, especially in vulnerable people such as those suffering from T1D.

From a clinical point of view, the present results are reassuring in that they indicate no noteworthy deterioration in terms of eating problems, but they also highlight how providing a diabetes care that meets patients’ needs is crucial, especially in a demanding time such as the COVID-19 pandemic. Transformations in daily routines and lifestyles—especially changes in meals, changes in schooling, lack of physical activity, and difficulties in accessing the health system due to the current pandemic—may in fact favor difficulties in properly managing diabetes and may negatively impact glycemic control. In order for individuals with T1D to not feel excessively burdened by their illness and its management, and in order to ensure overall health and wellbeing, continued appropriate and flexible medical, nutritional, and psychological care is essential (ADA, 2020; Delamater et al., 2018).

## Data Availability

N/A. N/A.

## Footnotes

1 Currently fully reimbursed by the Italian National Health Service (SSN), Regione Campania. The Italian SSN provides a uniform and comprehensive health system care covering the entire population, irrespective of individual ability to pay or other characteristics such as income or contributions, employment, or preexisting health conditions.
